# The Effects of Experimentally Induced Adelphophagy in Gastropod Embryos

**DOI:** 10.1371/journal.pone.0103366

**Published:** 2014-07-29

**Authors:** Olaf Thomsen, Rachel Collin, Allan Carrillo-Baltodano

**Affiliations:** 1 Smithsonian Tropical Research Institute, Balboa, Ancon, Republic of Panama; 2 Universität Oldenburg, Oldenburg, Germany; Australian Museum, Australia

## Abstract

Adelphophagy, development where embryos grow large by consuming morphologically distinct nutritive embryos or their own normal siblings is widespread but uncommon among animal phyla. Among invertebrates it is particularly common in some families of marine gastropods and segmented worms, but rare or unknown in other closely related families. In calyptraeid gastropods phylogenetic analysis indicates that adelphophagy has arisen at least 9 times from species with planktotrophic larval development. This pattern of frequent parallel evolution of adelphophagy suggests that the embryos of planktotrophic species might be predisposed to evolve adelphophagy. Here we used embryos of three species of planktotrophic calyptraeids, one from each of three major genera in the family (*Bostrycapulus*, *Crucibulum*, and *Crepidula*), to answer the following 3 questions: (1) Can embryos of species with planktotrophic development benefit, in terms of pre-hatching growth, from the ingestion of yolk and tissue from experimentally damaged siblings? (2) Does ingestion of this material from damaged siblings increase variation in pre-hatching size? and (3) Does this experimentally induced adelphophagy alter the allometry between the velum and the shell, increasing morphological similarity to embryos of normally adelphophagic species? We found an overall increase in shell length and velum diameter when embryos feed on damaged siblings within their capsules. There was no detectable increase in variation in shell length or velum diameter, or changes in allometry. The overall effect of our treatment was small compared to the embryonic growth observed in naturally adelphophagic development. However each embryo in our experiment probably consumed less than one sibling on average, whereas natural adelphophages often each consume 10–30 or more siblings. These results suggest that the ability to consume, assimilate, and benefit from yolk and tissue of their siblings is widespread across calyptraeids.

## Introduction

Marine invertebrates exhibit a remarkable variety of modes of development [1]–[3]. There are species with free-living larvae that swim and feed in the water column before they become competent to settle and metamorphose. There are species where large eggs develop directly into fully formed juveniles without the need for exogenous nutrition. Perhaps the most poorly understood mode of development is oophagic or adelphophagic development where embryos consume other eggs (oophagy) or embryos (adelphophagy), which provide exogenous nutrition prior to hatching (hereafter referred to simply as adelphophagy). Adelphophagic embryos usually develop directly into juveniles, bypassing the free-living larval stage. This results in large benthic hatchlings with low dispersal ability compared to those species with planktotrophic larvae. In this respect adelphophagic species are ecologically similar to species with direct development from large eggs. Why some species evolve direct development via large eggs and others evolve direct development via small eggs with adelphogphy is not clearly understood [4]–[5].

Encapsulated or brooded development where embryos develop in close proximity to their siblings is a prerequisite for the evolution of adelphophagy. Such protected development of early embryonic stages is common in many invertebrate groups, but the phylogenetic distribution of adelphophagy is patchy; being particularly common in some groups and absent in others. For example, among caenogastropods it is common in calyptraeids, muricids, vermetids, and buccinids but unknown in littorinids and conids, which also have encapsulated development [4], [6]–[10]. It is also particularly common in spionid polychaetes [11]. This suggests that the embryos of some groups may be predisposed to evolve adelphophagy.

Various consequences of adelphophagy have been demonstrated primarily in spionid worms and calyptraeid and muricid gastropods. In gastropods adelphophagy increases variation in hatching size [5], [7]–[8]. Several cases of poecilogony (an extreme case of variation in hatching size where females of a single species can produce more than one kind of development) result from adelphophagic development [12]. It remains to be seen if the increased variation in hatching size is a bet-hedging strategy or simply the by-product of a mechanism to produce large hatchlings [5]. In calyptraeid gastropods, adelphophagic development can, but does not always, produce hatchlings larger than those from large eggs [4]. Adelphophagic muricids may develop more quickly than do those with large eggs [6]–[7], however there is no evidence of rapid development in adelphophagic calyptraeids [5], and no other groups have been examined. In the adelphophagic worm *Boccardia proboscidia* females can actively control the time of hatching altering the size and developmental stage of hatchlings [13]. Genetic analyses have shown that adelphophagy increases the average relatedness of offspring within each capsule in one buccinid and one calyptraeid species [14]–[15]. Early in development the embryos within a capsule have more genetic diversity and represent offspring from more fathers than they do later in development. Despite these demonstrable consequences of adelphophagy, the factors that select for adelphophagic development remain unclear.

The developmental mechanisms by which nutritive eggs or embryos are specified, and the manner in which they are consumed by their siblings are still poorly understood. Nurse embryos or nurse eggs span a range of developmental potentials. In some cases they seem to have no potential to develop. For example the nurse eggs in the calyptraeid *Crepipatella dilatata* and the nassariid *Buccinanops globulosus* do not appear to be fertilized and do not initiate development [16]–[17]. In other species of calyptraeids, vermetids, and some spionid polychaetes the nurse embryos initiate development but become arrested at some point prior to the development of the definitive juvenile body plan [18]–[23]. Finally, in the polychaete *Boccardia proboscidia*, the wormsnail *Vermetus triquetrus*, and another unidentified vermetid some well-developed, fully-functional embryos are consumed by their siblings [13], [23]–[24]. In none of these species is it known if the arrested embryos have the potential to develop into normal hatchlings if some environmental factor did not arrest their development, or, alternately if their developmental fate is specified prior to ovulation or oviposition.

The mechanisms by which developing embryos consume nutritive embryos vary as widely as their appearance. In some species the nutritive eggs or embryos are swallowed whole by their siblings. This is often the case in species where the nutritive eggs do not cleave, like in *Buccinum undatum* [25]. Consumption of entire embryos has also been observed in this species [25]. In other cases blobs of yolk are detached from the surface or sucked from the interior of the nutritive embryos [8]–[9], [17], [19]–[22]. This seems to be particularly common in calyptraeids where species of *Crepidula*, *Calyptraea*, *Crepipatella,* and *Crucibulum* have all been described has having nutritive embryos that either disintegrate or become hollowed out as development progresses [19], [20], [26]. In these species nutritive embryos may follow developmental pathways that produce yolk vesicles that are easily detached and consumed by their normal siblings [26]. The role of apoptosis in producing such vesicles has been demonstrated in the polychaete *Polydora cornuta* [21] but has not been looked for in adelphophagic gastropods.

In families were adelphophagy is common it appears to have arisen multiple times. For example in calyptraeid gastropods phylogenetic analysis indicates that adelphophagic or oophagic development has arisen at least 9 times, generally from species with planktotrophic larval development [18], [27]. The developing embryos in many adelphophagic calyptraeid species do not appear to be modified from the typical development of their planktotrophic relatives, other than having a relatively larger shell and visceral mass [18]–[20], [26], [28]–[30]. This suggests that the evolution of adelphophagy is relatively simple in this family, and that the embryos of related planktotrophic species might already express features that enable them to take advantage of dead or damaged siblings. Such “pre-adaptations” for adelphophagy could involve both the ability to capture and ingest yolk particles within the egg capsule and the digestion, absorption, and utilization of the material for growth and development.

Here we used three species of planktotrophic calyptraeids, one from each of three major genera in the family (*Crepidula*, *Crucibulum*, and *Bostrycapulus*) to answer the following 3 questions: (1) Can embryos of species with planktotrophic development benefit, in terms of pre-hatching growth, from the ingestion of yolk and tissue from experimentally damaged siblings? (2) Does ingestion of yolk and tissue from siblings increase variation in pre-hatching size? and (3) Does this experimentally induced adelphophagy alter the allometry between the velum and the shell, increasing morphological similarity to embryos of normally adelphophagic species? Anecdotal accounts report that in species with planktotrophic development the occasional empty shell is found in egg capsules containing otherwise normally developing embryos [27], [31]. This suggests that embryos occasionally die naturally during development and that their siblings may consume the tissue. Such consumption of dead siblings could even be selected for if it prevented contamination of the egg capsule. In addition, embryos of *Crepipatella peruviana* (previously referred to by the name *Crepidula fecunda* or *Crepipatella fecunda* see [32] for a discussion of taxonomic revisions in *Crepipatella*), a species that is thought to have recently re-evolved planktotrophic development from oophagy can consume damaged siblings prior to hatching, and that this can lead to increased hatching size [31], [28]. Our study was designed to determine if this capacity is common among primary planktotrophs across the family, thus setting the stage for the evolution of adelphophagy.

## Materials and Methods

Snails were collected under permits issued by the Autoridad de Recursos Acuáticos de Panamá (ARAP). *Crepidula* cf. *marginalis* were collected at Chumical Beach (8° 53′ N, 79° 38′ W) and *Bostrycapulus calyptraeformis* (Deshayes 1830) and *Crucibulum spinosum* (Sowerby 1824) were collected at Venado Beach (8° 53′ N, 79° 35′ W), near Panama City, Panama. All 3 species occur in the intertidal with a mix of soft bottom and rocky-rubble, and were collected between June and August 2012. All three species have planktotrophic development with similar ranges of hatching sizes; *Bostrycapulus calyptraeformis* hatches at 300–345 µm shell length; *Crucibulum spinosum* hatches at 280–325 µm shell length and *Crepidula* cf. *marginalis* hatches at 290–330 µm shell length [4], [33]. In all 3 species the female produces clutches of multiple transparent thin-walled capsules, which she protects between the substrate, her propodium, and her neck. Each capsule contains numerous (50–150) small eggs, each of which normally develops into a veliger larva. The embryos are free within the capsule, and shortly after gastrulation they can be seen to move actively around the capsule, often bumping into and pushing past each other. When each mature veliger no longer has visible yolk, the larvae hatch with the help of the mother. In calyptraeid species where this has been observed, the female appears to nudge the capsules out from under the shell, sometimes pulling at them with her mouth, while pumping the shell up and down [30]. This presumably acts to disperse the larvae away from the substrate. Capsules raised in still culture away from the mother do not hatch. Instead the larvae continue to swim actively inside the capsule well past their due date, and eventually they appear to starve [26], [30].

Large females were collected and kept individually at room temperature, approximately 22–25°C, in 350 ml transparent plastic cups filled with filtered, UV-sterilized seawater. The water was changed three times a week, and the snails were fed approximately 38.6×10^6^ cells of *Isochrysis galbana* daily (following [34]). Females were monitored and the date of egg deposition was recorded. Egg capsules were collected from brooding females after 7 or 8 days of incubation, when they were at a stage capable of ingesting exogenous particles. This “head vesicle stage” is characterized by the extension of the velum away from the body wall and the presence of a transparent, ciliated head vesicle (see [35]). It is at this stage that many naturally adelphophagic calyptraeid species begin the consumption of nurse eggs. Preliminary attempts to apply our experimental manipulations to embryos prior to the head-vesicle stage generally resulted in the fouling of the egg capsules and infection with microorganisms. The head vesicle stage is approximately 4–5 days before hatching would normally be induced by the female. Once this stage was reached capsules were taken from the female, and placed in 40×12 mm Petri dishes containing 0.22 µm-filtered, UV-sterilized seawater and antibiotics (5.2×10^−5^ g/ml Penicillin G potassium salt and 9.2×10^−4^ g/ml streptomycin sulfate salt) to reduce contamination from microorganisms [36].

**Figure 1:**
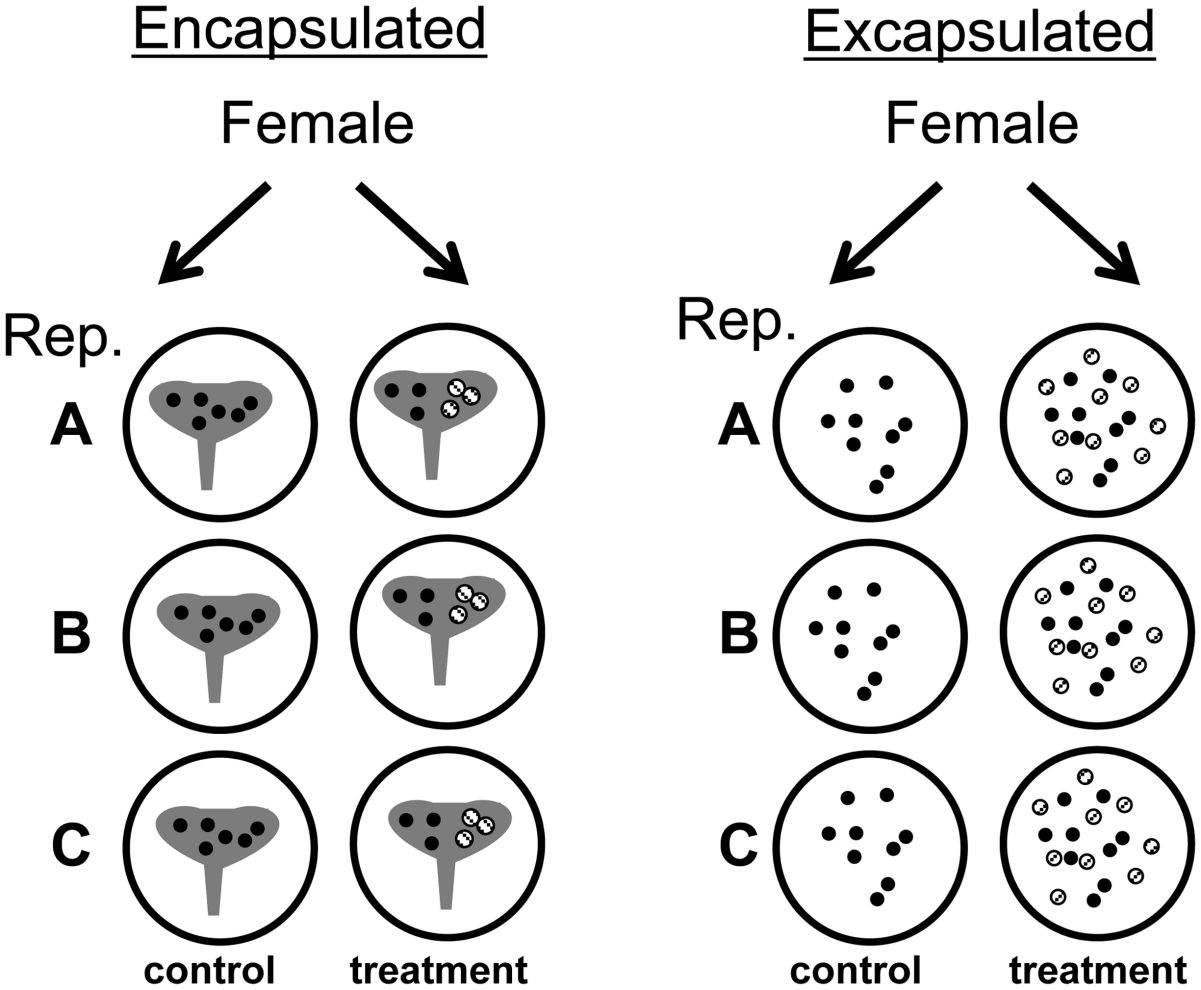
Experimental protocol for the Encapsulated and Excapsulated experiments. Capsules were placed into 6 replicate dishes, 3 assigned to the experimental treatment and 3 to the control. Solid black circles indicate embryos and hashed circles indicate embryos that were smashed.

**Figure 2:**
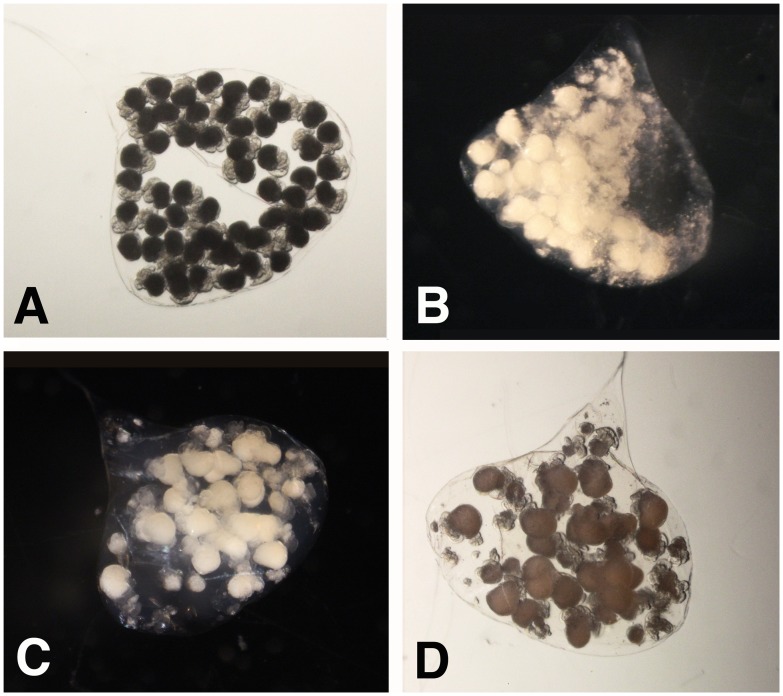
Yolk consumption of *Crepidula* cf. *marginalis* in the Encapsulated experiment. **A**. Control embryos in an untreated capsule. **B**. Embryos in the capsule immediately after the manipulation that smashed some of the embryos. **C**. and **D**. Experimental capsules 1.5 hours after the treatment. The fine yolk particles have been cleared from the capsule and only some larger particles, most of which will disintegrate over the next few days, remain.

**Figure 3:**
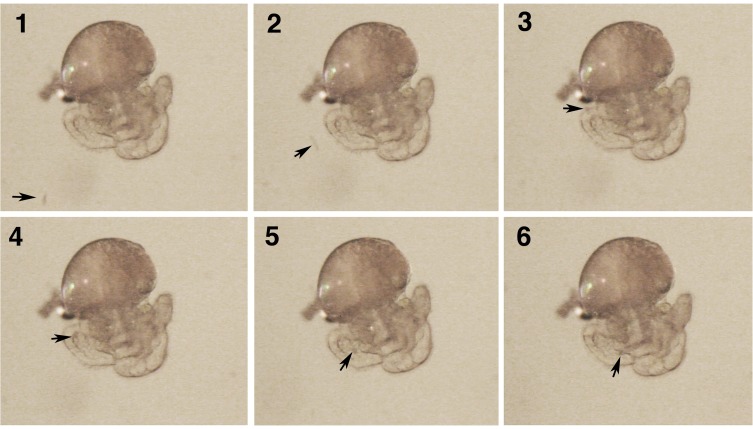
A series of video frames of a freshly excapsulated *Crepidula* cf. *marginalis* embryo capturing a small particle of yolk marked by the arrow. The particle approaches the embryo, is caught on the margin of the velum and is transported along the food groove to the mouth.

**Figure 4:**
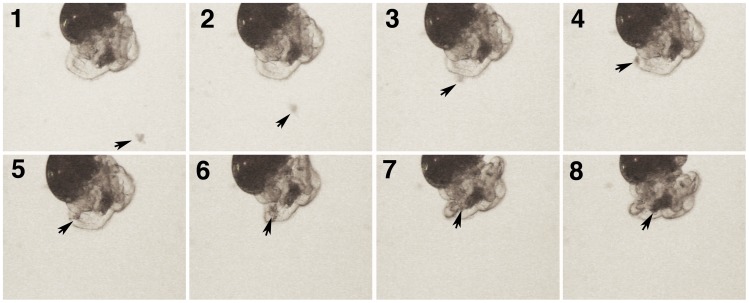
A series of video frames of an excapsulated *Crepidula* cf. *marginalis* embryo after it has fed for 40 minutes. The anterior opaque area shows yolk accumulated in the esophagus and buccal cavity. In this series a larger particle is transported via a combination of cilia on the food groove and a twitch of the velum, and is added to the yolk accumulated at the mouth.

For the *Encapsulated Experiment* 6 capsules were removed from the female and each was placed individually in a Petri dish. Three capsules were assigned to the treatment and 3 were assigned to the control (Figure 1). For treated capsules 20–40% of the embryos within a single capsule were killed by applying pressure to the intact capsule with thin flat forceps (Figure 2). Care was taken not to rupture the capsule wall, and any capsules that appeared damaged were discarded. Observation of the treated capsules showed that the small yolk particles were cleared from the capsule fluid within 2–3 hours and that larger fragments took longer to clear (Figure 2). Because the capsules are a closed system, and because undamaged embryos were observed to capture and ingest yolk particles (Figures 3 & 4, Video S1), it seems likely that this material was ingested. For the control group, the intact capsules were each left in a Petri dish without killing any of the embryos. Broods from 9 females were used for *C.* cf. *marginalis*, 11 for *C. spinosum*, and 16 for *B. calyptraeformis*.

For the *Excapsulated Experiment* three replicates were used from each of 12 mothers for *C.* cf. *marginalis* only. In the ‘treated’ group, two capsules were placed into the Petri dish (Figure 1). The contents of one capsule was killed completely and emptied into the Petri dish, while the other capsule was opened so that the embryos swam freely and could feed on their dead siblings. The embryos were allowed to feed for one hour (Figures 3 & 4), and were then transferred to a new Petri dish with fresh filtered seawater. In the control group, the embryos of one capsule were released into the Petri dish without an additional food source.

All dishes were maintained in an incubator at 24°C and the water was exchanged daily, with new filtered seawater and antibiotics. To measure the velum and shell length, embryos were removed from the capsules and photographed three days after the treatment was imposed, slightly before the date on which the embryos were due to hatch. We have never observed capsules of these species to hatch naturally when they have been removed from the mother, so it was not possible to determine if the treatments had an effect on natural hatching. Lateral views of the embryos were used to measure the shell length, and anterior views were used to measure the diameter across the two extended velar lobes using ImageJ 1.46r [37]. Each of these measurements was taken for at least 10 embryos from each replicate dish. A two-level nested ANOVA was used to test for effects of treatment, female, and treatment × female on larval morphology, using JMP 9.0.2. The replicate dishes were included as a random effect nested within female. To test for changes in the allometry between shell length and velum diameter we used an ANCOVA with average velum diameter for each replicate as the dependent variable, treatment as a factor, and average shell length for the same replicate as a covariate. We used average values for each replicate because the shell length and velum size measurements often could not be obtained for the same individual embryo. To test for differences in variation between treated and control embryos the coefficient of variation (CV) was calculated for each replicate dish. ANOVA was used to test for the effect of female, treatment, and the interaction between female and treatment on the CV of shell length and CV of velum diameter.

## Results

### Encapsulated Experiment

In general capsules from *Crepidula* cf. *marginalis* responded the best to the experimental treatment. They were seldom infected with microorganisms and the development of most embryos appeared normal. *Crucibulum spinosum* and *Bostrycapulus calpytraeformis* suffered from infections somewhat more frequently and capsules occasionally contained unusual yolky blobs (*C. spinosum*) or poorly developed embryos. This was true for both treated and control capsules. We therefore, limited the excapsulated treatment, which seemed more likely to suffer from infection, to *C*. cf. *marginalis*. Overall 847 embryos were measured from encapsulated *C.* cf. *marginalis*, 1228 from *Crucibulum spinosum* and 1928 from *Bostrycapulus calyptraeformis*.

**Figure 5:**
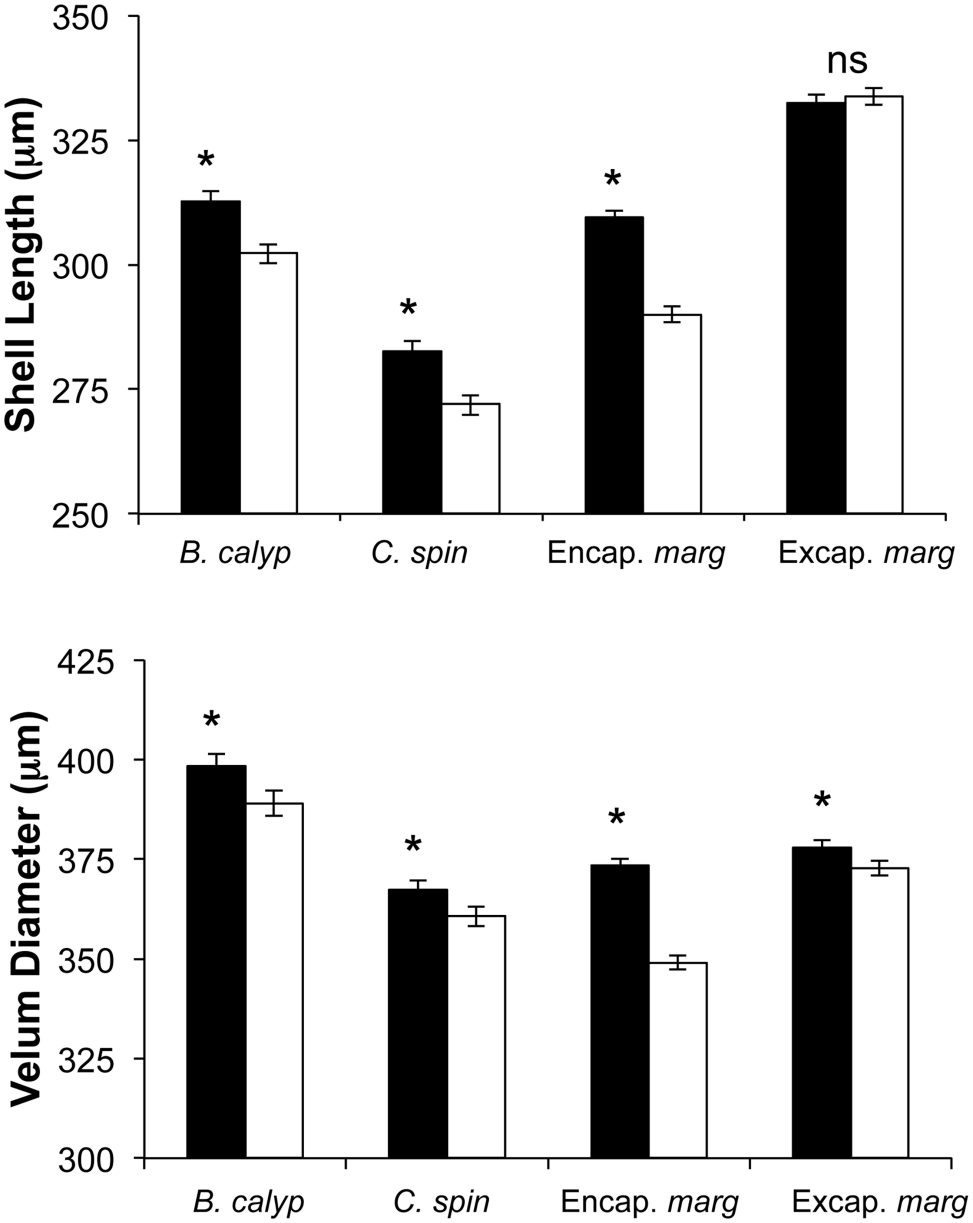
Bar graph showing the effect of the Encapsulated and Excapsulated experiments on shell length and velum diameter of encapsulated *Bostrycapulus calyptraeformis* (*B. calyp*), encapsulated *Crucibulum spinosum* (*C. spin*), encapsulated *Crepidula* cf. *marginalis* (Encap *marg*), and excapsulated *Crepidula* cf. *marginalis* (Excap *marg*). * indicates statistical significance at the P<0.05 level (see Tables 1 & 3 for details). Mean shell lengths for encapsulated embryos (treated vs. controls): *B. calyptraeformis*: 312.70 µm (s.e.  = 1.98) versus 302.37 µm (s.e.  = 2.07); *C. spinosum*: 282.55 µm (s.e.  = 2.19) versus 272.04 µm (s.e.  = 2.18); *C.* cf. *marginalis*: 309.42 µm (s.e.  = 1.46) versus 287.93 µm (s.e.  = 1.46). Mean velum diameters for encapsulated embryos (treated vs. controls): *B. calyptraeformis*: 398.78 µm (s.e.  = 7.78) versus 380.80 µm (s.e.  = 7.81); *C. spinosum*: 367.24 µm (s.e.  = 2.44) versus 360.66 µm (s.e.  = 2.44); *C.* cf. *marginalis*: 373.44 µm (s.e.  = 1.72) versus 349.10 µm (s.e.  = 1.74).

**Figure 6:**
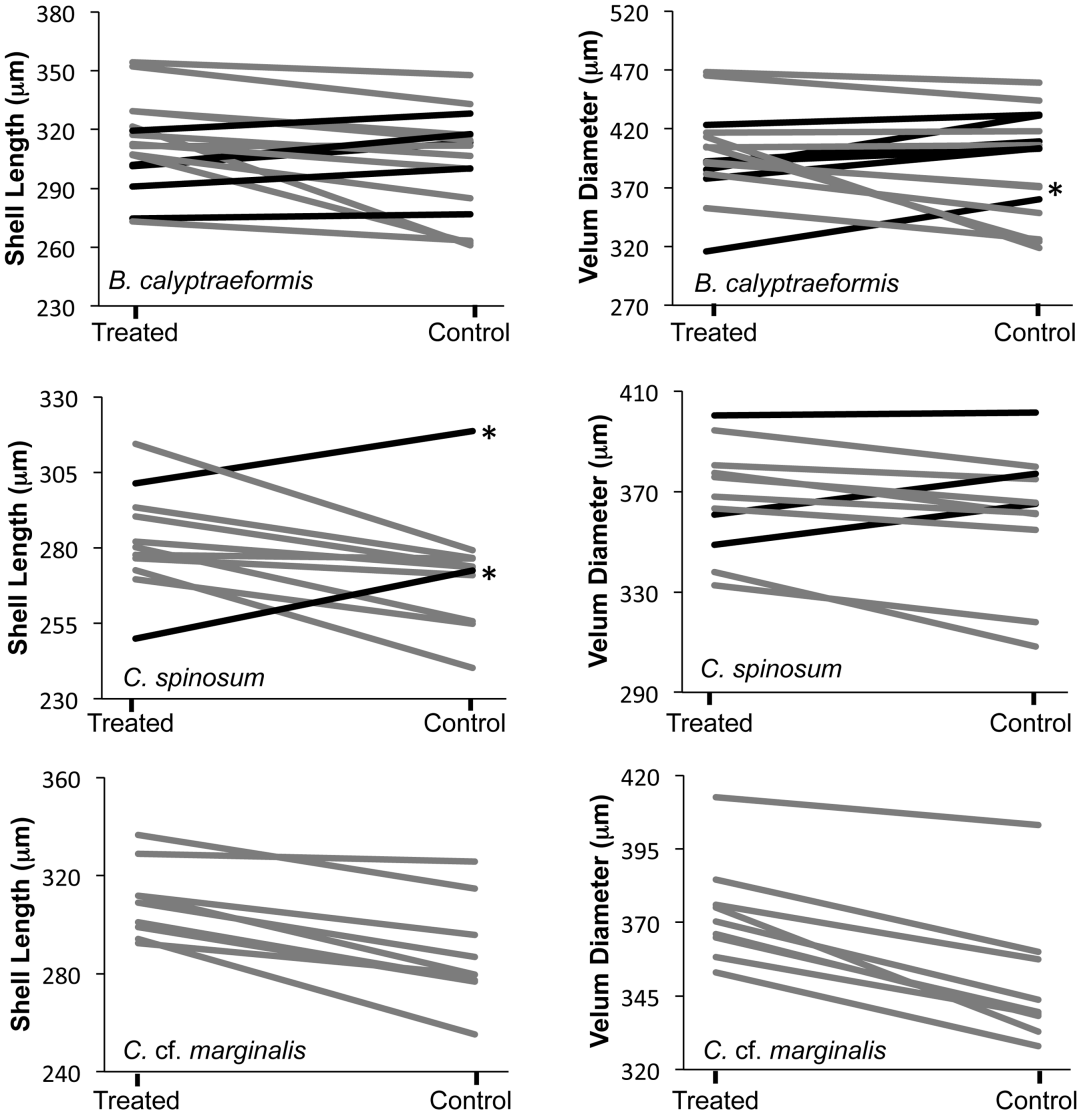
The norms of reaction for each female, showing the effect of treatment on average shell length and average velum diameter for broods from each female in the Encapsulated experiment. Gray lines show broods for which the treated embryos were larger than the controls and black lines show the individuals for which the opposite pattern was observed. * indicates those broods where the post-hoc test shows a significant smaller size in the experimental embryos compared to the controls at P<0.05 level.

Shell length and velum diameter were both significantly larger in treated embryos compared to control embryos in all three species (Figure 5; Table 1). Mean shell lengths were between 3% and 7% larger in treated embryos than in controls and velum diameters were between 2% and 7% larger in treated embryos than in controls (Figure 5). In all three there were significant effects of female and a significant interaction between female and treatment (Table 1; Figure 6). The random effect of replicate nested within female explained 19–38% of the variation in shell length, and 7–26% of the variation in velum diameter. This is most likely due to a combination of slight differences in egg size between capsules and differences in the number of embryos that were successfully smashed in our experimental treatment.

**Table 1:** Results from the ANOVA of shell length and velum diameter of Encapsulated embryos.

	Shell Length				Velum Diameter			
Effect	df	F	P	% var	df	F	P	% var
*Bostrycapulus calyptraeformis*								
Treatment	1	83.52	**<0.0001**		1	14.43	**0.0002**	
Female	15	9.37	**<0.0001**		15	10.17	**<0.0001**	
Female × treatment	15	36.96	**<0.0001**		15	21.79	**<0.0001**	
Replicate [female] random	-	-	**-**	38.01	-	-	**-**	26.41
*Crucibulum spinosum*								
Treatment	1	129.48	**<0.0001**		1	14.90	**0.0001**	
Female	10	5.07	**0.0007**		10	9.46	**<0.0001**	
Female × treatment	10	37.46	**<0.0001**		10	6.99	**<0.0001**	
Replicate [female] random	-	-	**-**	37.39	-	-	**-**	15.98
*Crepidula* cf. *marginalis*								
Treatment	1	486.76	**<0.0001**		1	210.50	**<0.0001**	
Female	8	18.66	**<0.0001**		8	19.37	**<0.0001**	
Female × treatment	8	13.77	**<0.0001**		8	3.76	**0.0003**	
Replicate [female] random	-	-	**-**	18.91	-	-	**-**	7.13

Statistically significant results (P<0.05) are highlighted by bold font.

In *Crepidula* cf. *marginalis* the significant interaction between female and treatment was due to differences in slopes in the reaction norms for each female; but the response to the treatment was in the same direction for all females (Figure 6). For *C. spinosum* two females had a significantly larger shell length in the controls compared to the treated embryos (black lines in Figure 6; post-hoc Tukey's HSD test P<0.05). This response was evident in all of the replicates for these females. However, it was observed that the treated capsules were full of a yolky emulsion when they were opened. For some reason the embryos from these females may not have been able to clear the capsules of the particles of dead siblings which may have inhibited their natural growth compared to controls. Alternately the remaining embryos themselves may have been disintegrating for some reason. This emulsion was not seen in any of the treated capsules that produced embryos with larger shell lengths or velar diameters compared to their controls. Three *C. spinosum* females, including the two with unusual yolky capsular fluid, also had larger average velum diameters in the controls than the treated embryos (Figure 6). None of these comparisons were significant in the post hoc test (P>0.05). For *Bostrycapulus calyptraeformis* the response of each female to the treatment was less consistent than in the other two species. Here, despite an overall significant increase in shell length and velum diameter in the treated embryos, 5 females showed smaller shell lengths in treated capsules (none of which were significant at P<0.05 in Tukey’s HSD post-hoc tests) and 7 females showed smaller velum diameters (one of which was significant in Tukey's HSD post-hoc tests). These non-significant post-hoc comparisons suggest that the significant interaction term is due to a lack of a significant response to the treatment in a minority of the females, rather than differences between females in the direction of significant responses.

**Figure 7:**
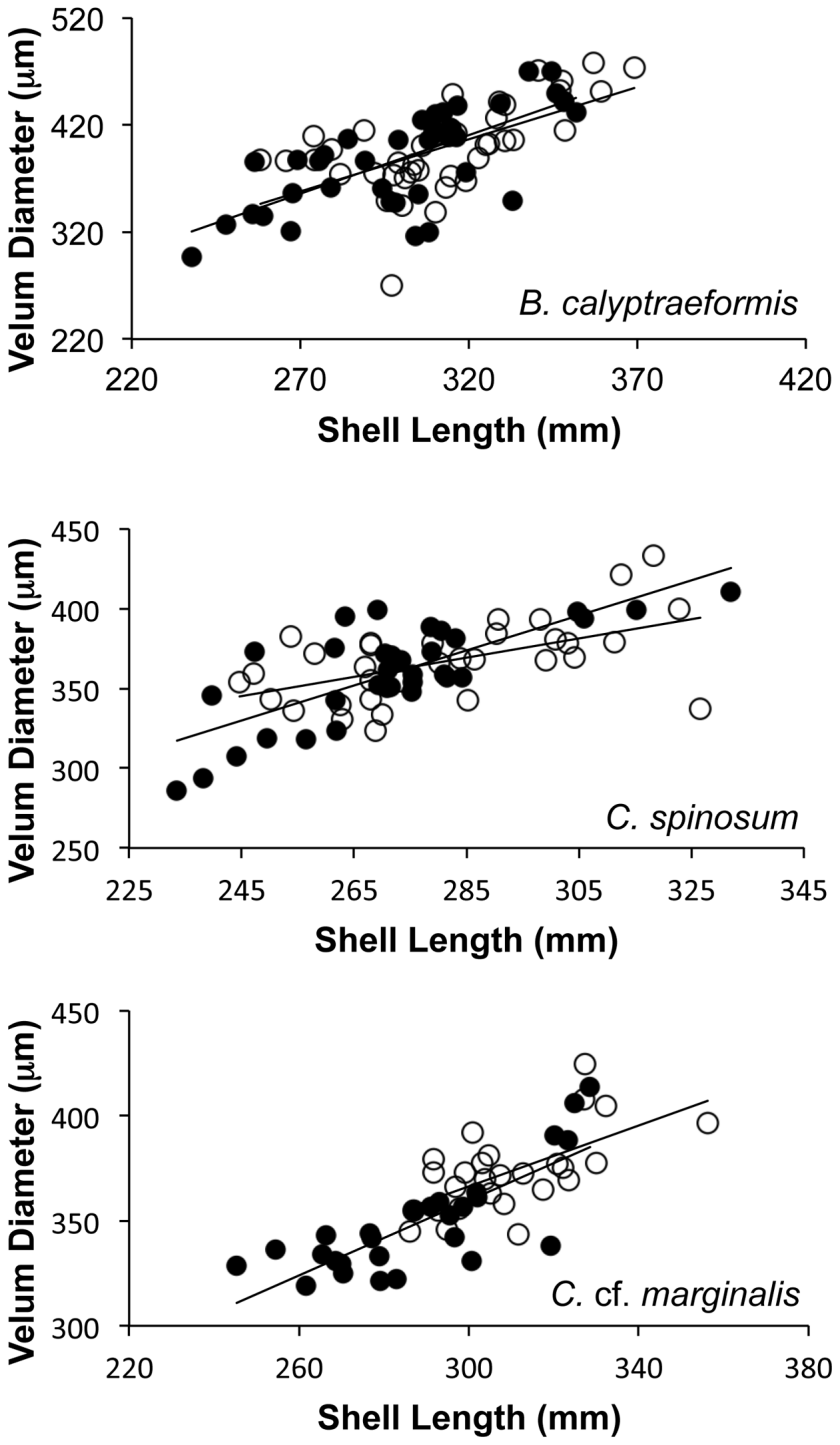
Effect of treatment on allometry between average shell length and average velum diameter in treated and control dishes. There were no significant effects of treatment on allometry in any species when the two females with yolky emulsion in their capsules were removed from the analysis of *C. spinosum* (see text for details).

The experimental treatment did not alter the overall allometry between the average velum diameter and average shell length in *Bostrycapulus calyptraeformis* or *Crepidula* cf. *marginalis* (Figure 7). ANCOVA showed that in all three species velum diameter was positively correlated with shell length (P<0.0001) but was not affected by treatment (P = 0.75; P = 0.95; P = 0.16; *B. calyptraeformis*, *C. spinosum* and *C.* cf. *marginalis* respectively). The lack of an effect of treatment on velum size in this ANCOVA is due to smaller sample sizes and low power when individuals are averaged within each replicate dish, in contrast to the significant effect recovered by the nested ANOVA above. The ANCOVA recovered no effect of the interaction between treatment and shell length on velum size for *B. calyptraeformis* (P = 0.66) or *C.* cf. *marginalis* (P = 0.47). The interaction was significant for *C. spinosum* (P = 0.042), where the velum size increased more slowly with increasing shell length in treated embryos than in control embryos (Figure 7). This relationship disappeared when the two females with yolky emulsion in the treated capsules were removed from the analysis.

The experimental treatment did not increase the variability of shell length or velum diameter for *B. calyptraeformis* or *C. spinosum* (Table 2). There was no significant effect of treatment nor a significant interaction between female and treatment on the coefficient of variation in shell size or velum diameter. One female *B. calyptraeformis* had unusually variable embryos and her 3 replicate control capsules were identified as outliers from visual inspection of the residuals plot. When she was included in the analysis there was a significant interaction between female and treatment for that species (P = 0.015). When her broods were removed from the analysis this effect was no longer significant (Table 2). In *C.* cf. *marginalis* there was an unexpected significant effect of treatment on variation in shell length. Treated embryos had significantly less variation (CV  = 3.64) in shell length than control embryos (CV  = 4.34).

**Table 2:** Results of ANOVA on coefficient of variation of shell length and velum diameter for each replicate dish of encapsulated embryos.

	Shell Length			Velum Diameter		
Effect	df	F	P	df	F	P
*Bostrycapulus calyptraeformis*						
Treatment	1	0.01	0.26	1	0.62	0.44
Female	13	4.21	**<0.005**	14	5.89	**<0.0001**
Female × treatment	13	1.55	0.65	14	0.76	0.54
*Crucibulum spinosum*						
Treatment	1	0.76	0.38	1	0.25	0.62
Female	10	2.01	0.06*	10	1.79	0.09*
Female × treatment	10	1.57	0.15	10	0.69	0.73
*Crepidula* cf. *marginalis*						
Treatment	1	3.73	**0.06** #	1	1.32	0.26
Female	8	2.81	**0.02**	8	2.71	**0.02**
Female × treatment	8	0.63	0.74	8	0.67	0.72

Statistically significant results (P<0.05) are highlighted by bold font.

*These factors do not become significant with the removal of the non-significant interaction term.

# This factor becomes significant with the removal of the non-significant interaction (P = 0.009).

### Excapsulated Experiment

Embryos subjected to the excapsulated treatment often became contaminated with microorganisms, resulting in the abnormal development of the embryos. However replicate broods with normal-appearing embryos were measured for 12 females. For these there was no significant effect of treatment on shell length, but velum diameter was significantly larger in treated than in control embryos (Table 3; Figure 5). For both velum diameter and shell length there were significant effects of female or the interaction between female and treatment. The random effect of replicate dish accounted for 11% of the variation in velum diameter and 34% in shell length. Treatment did not alter the allometry between shell length and velum diameter (ANCOVA: treatment × shell length P>0.05). Again the power to detect a difference was reduced by the use of means for each dish.

**Table 3:** Results from the ANOVA on shell length, velum diameter, and coefficient of variation of each from Excapsulated embryos of *Crepidula* cf. *marginalis*.

	Shell Length				Velum Diameter			
Effect	df	F	P	% var	df	F	P	% var
Treatment	1	2.51	0.11		1	9.21	**0.0025**	
Female	11	18.50	**<0.0001**		15	22.48	**<0.0001**	
Female × treatment	11	24.34	**<0.0001**		15	7.47	**<0.0001**	
Replicate [female] random	-	-	-	33.56	-	-	-	11.24

	CV Shell Length			CV Velum Diameter		
Effect	df	F	P	df	F	P
Treatment	1	11.73	**0.001**	1	0.05	0.82
Female	11	10.39	**<0.0001**	11	4.05	**0.0004**
Female × treatment	11	3.22	**0.003**	11	0.55	0.86

Statistically significant results (P<0.05) are highlighted by bold font.

There was a significant effect of treatment (P<0.005) and female (P<0.001), and of the interaction between female and treatment (P<0.005) on CV in shell length. Treated embryos were more variable (CV  = 4.08) than control embryos (CV  = 3.15). The significant effect of treatment was due entirely to the broods from two females (with CV  = 9.75 and 7.13 for treated embryos). One of these had significantly smaller and less well-developed embryos than any other female. It is possible that the treatment was applied at a slightly earlier developmental stage for this brood. Treatment had no significant effect on the CV of velum diameter.

## Discussion

Our results and previously published data on *Crepipatella peruviana* [31] indicate that the ability to consume, absorb, and utilize material derived from dead and damaged siblings occurs in embryos of planktotrophic calyptraeids from 4 genera. Destruction of 20–40% of the embryos within a capsule resulted in a statistically significant increase in shell length and velum diameter in the three species studied here. A similar result was also obtained for some embryos of *Crepipatella peruviana*, but the results of this previous study were mixed [31]. Embryos in that study which took a particularly long time to hatch showed a significant increase in shell length and velum area. Those that hatched particularly quickly showed the opposite effect, and those that hatched after the average duration showed no effect of the treatment. There were a number of differences between the protocols of these studies, which could account for the somewhat different results. Our study used tropical species raised in petri dishes with antibiotics, and embryos were measured a standard number of days after the experimental treatment was imposed. Cubillos et al. [31] used a temperate species, the capsules were raised in aquaria without antibiotics, and the capsules were allowed to hatch naturally, resulting in significant variation in the time between when the treatment was imposed and when the larvae were measured. Despite these differences in protocol, it is clear that like the species studied here, embryos of *Crepipatella peruviana* can ingest yolk and tissue from their damaged siblings and, at least in some cases, this material is used to support additional growth and development [31].

Another difference between our two experimental designs was the way that differences among females and broods were accounted for. Differences among the broods from each female and even broods from the same female can be an important source of variation in offspring size in calyptraeids, often accounting for almost half of the observed variation [5], [33], [38]. For all three species studied here, a statistically significant effect of “female” was recovered in virtually all of the tests. We used only a single brood from each female and all of the broods were not collected from them at exactly the same moment in development. Therefore this “female” effect incorporates a combination of variation among females, among broods from the same female which may differ in egg size or quality, as well as any differences in the exact stage at which the experimental treatment was imposed. Differences between capsules from the same clutch, variation due to our inability to exactly replicate the smashing treatment, as well as any dish effects were accounted for by the random effect of replicate. The experimental design of Cubillos et al. [31], which did not explicitly take into account this variation, may have had reduced power to detect the significant effects of the treatments if “female” effects account for a large proportion of the variance in *C. peruviana*. An intriguing suggestion of their results is that embryos exposed to the treatment earlier (i.e., longer time to hatch) respond differently to the availability of yolky material than do embryos that are exposed later in development. It would be interesting to test this possibility explicitly.

The magnitude of the effects of consuming siblings detected here were modest. In *C. peruviana* a 16% average increase in shell length was detected in broods that showed an increase in size in treated embryos. In the present study, however, the average shell lengths of experimental embryos only exceeded those of controls by 3–7%. In neither study did the shell length of artificially adelphophagic embryos approach the length at hatching of species that are naturally adelphophagic, nor did we expect them to. Because 20%–40% of the embryos in each capsule were killed, we expected each remaining embryo to consume less than the material from a single sibling. In normally adelphophagic species the number of nurse embryos usually exceeds the number of embryos that will complete development by an order of magnitude. For example in *Crepipatella dilatata*, the sister species to *C. peruviana*, each developing embryo consumes between 15 and 35 uncleaved nurse eggs to produce a 1–1.8 mm hatchling [39]. In addition, in our experiment, we opened the capsules 3 days after the experimental treatment was imposed, while the embryos still retained some little yolk. It is possible that embryos that had ingested material from siblings could have continued growth and development for longer than controls and ultimately demonstrated a larger effect. Our standardized protocol could not detect this possible greater potential for growth of the treated embryos, because capsules of these species do not hatch naturally away from the mother. Even if the conversion of ingested material to embryonic growth is not as efficient in normally planktotrophic embryos as in embryos that normally utilize nurse eggs or embryos, providing a greater number of damaged siblings, over a longer span of development than used here might produce a larger effect similar to Cubillos et al. [31]. If a larger effect could be produced it would be possible to examine the effects of changes in size induced by this treatment on larval growth and survival.

We did not observe changes in the variation or morphology of the embryos that indicated increased similarity to species with adelphophagic development. It is well documented that one of the consequences of adelphophagy is an increased variation in hatchling size within and among females [5]. Our treated embryos did not generally show a significant increase in variation in either shell length or velum diameter. This could be due to the overall small effect of the treatment. A treatment with a larger absolute effect on embryo size might have produced a more easily detectable change in variation. We also failed to detect significant differences in shell-velum allometry in treated and control embryos. We had predicted that increased energy from consumption of siblings would reduce the velum size relative to shell length in treated embryos. This prediction was based on two observations. It is common for embryos of both adelphophagic species and species that develop from large yolky eggs to have velar lobes that are smaller relative to the shell length than in planktotrophic species ([18]; Collin per. obs.). Also, changes in shell-velum allometry have been induced by different food rations in planktotrophic mollusc larvae, with well-fed larvae having relatively smaller velar lobes than less well-fed larvae [40], [41]. We failed to find a change in allometry in response to consumption of dead siblings, instead it appeared that the increase in resources resulted in a uniform increase in size. The small overall effect of our treatment and the use of dish averages make it difficult to strongly demonstrate that there was no change in allometry

Our results suggest that planktotrophic calyptraeid embryos typically express features that enable them to take advantage of abnormally developing embryos, and that embryologically the evolution of adelphophagy might be fairly easy. Head vesicle stage embryos of most planktotrophic species have ciliation on the velum and head vesicle (e.g., [42]) that can be used to capture small particles [28], [41]. Assays have shown that head vesicle stage embryos of *Crepipatella peruviana* and diverse other calyptraeids can ingest 2 µm plastic beads [28] (R. Collin unpublished data). The cilia of the velum and sometimes the uniform ciliation of the head vesicle can sweep various kinds of debris into the mouth in embryos of several planktotrophic *Crepidula* and *Bostrycapulus* species (Figures 3 & 4; R. Collin pers. obs.). It seems likely from these observations that the ability to capture particles in pre-hatching stages of development is widespread among planktotrophic calyptraeids. What is less well known is the ability of these embryos to absorb and utilize the material they capture. The results of the present study and [31] demonstrate that all species that have been examined to date can grow in response to ingesting particles of their siblings. This ability may be widespread in calyptraeids, and may mean that the evolution of adelphophagy does not require any special embryological modifications, explaining the frequent parallel evolution of adelphophagy in this family. It would be interesting to conduct similar experiments in vermetids and muricids, where adelphophagy is common, to determine if the ability to consume and utilize damaged siblings is common across families. Comparisons with a group that lacks adelphophagy entirely could be used to determine if constraints on the ability of normal embryos to benefit from the ingestion of siblings inhibit the evolution of adelphophagy in those groups.

## Supporting Information

Video S1 *Crepidula* cf. *marginalis* embryos capturing particles. This video shows several sequences of embryos using the cilia on the velum to capture yolk particles from damaged siblings.
(M4V)
